# The Chemokine Receptor CCR8 Is a Target of Chimeric Antigen T Cells for Treating T Cell Malignancies

**DOI:** 10.3389/fimmu.2022.808347

**Published:** 2022-05-26

**Authors:** Diwei Zheng, Xindong Wang, Lin Cheng, Le Qin, Zhiwu Jiang, Ruocong Zhao, Yao Li, Jingxuan Shi, Qiting Wu, Youguo Long, Suna Wang, Zhaoyang Tang, Wei Wei, Jie Yang, Yangqiu Li, Hongsheng Zhou, Qifa Liu, Pentao Liu, Xinwen Chen, Yao Yao, LiHua Yang, Peng Li

**Affiliations:** ^1^China-New Zealand Joint Laboratory of Biomedine and Health, State Key Laboratory of Respiratory Disease, Guangdong Provincial Key Laboratory of Stem Cell and Regenerative Medicine, Chinese Academy of Sciences Key Laboratory of Stem Cell and Regenerative Medicine, Guangzhou Institutes of Biomedicine and Health, Chinese Academy of Sciences, Guangzhou, China; ^2^University of Chinese Academy of Sciences, Beijing, China; ^3^Bioland Laboratory (Guangzhou Regenerative Medicine and Health Guangdong Laboratory), Guangzhou, China; ^4^Centre for Regenerative Medicine and Health, Hong Kong Institute of Science and Innovation, Chinese Academy of Sciences, Hong Kong, Hong Kong SAR, China; ^5^Guangdong Zhaotai In vivo Biomedicine Ltd., Guangzhou, China; ^6^Guangdong Zhaotai Cell Biology Technology Ltd., Foshan, China; ^7^Guangdong Cord Blood Bank, Guangzhou, China; ^8^Guangdong Women and Children Hospital, Guangzhou, China; ^9^Department of Hematology, First Affiliated Hospital, Jinan University, Guangzhou, China; ^10^Department of Hematology, Nanfang Hospital, Southern Medical University, Guangzhou, China; ^11^School of Biomedical Sciences, Stem Cell and Regenerative Medicine Consortium, Li Ka Shing Faculty of Medicine, The University of Hong Kong, Pok Fu Lam, Hong Kong SAR, China; ^12^Department of Pediatric Hematology, Zhujiang Hospital, Southern Medical University, Guangzhou, China

**Keywords:** CCR8, TAX, ATLL, T cell malignancy, CAR T cells

## Abstract

Chimeric antigen receptor (CAR) T cells have been successfully used in the therapy of B cell leukemia and lymphoma, but still have many challenges in their use for treating T cell malignancies, such as the lack of unique tumor antigens, their limitation of T cell expansion, and the need for third party donors or genome editing. Therefore, we need to find novel targets for CAR T cell therapy to overcome these challenges. Here, we found that both adult T-cell leukemia/lymphoma (ATLL) patients and ATLL cells had increased CCR8 expression but did not express CD7. Moreover, targeting CCR8 in T cells did not impair T cell expansion *in vitro*. Importantly, anti-CCR8 CAR T cells exhibited antitumor effects on ATLL- and other CCR8-expressing T-ALL cells *in vitro* and *in vivo*, and prolonged the survival of ATLL and Jurkat tumor-bearing mouse models. In conclusion, these collective results show that anti-CCR8 CAR T cells possess strong antitumor activity and represent a promising therapeutic approach for ATLL and CCR8^+^ tumors.

## Introduction

Although chimeric antigen receptor (CAR) T cell therapies lead to high clinical response rates in patients with certain B cell malignancies (1, 2), their use for treatment of T cell malignancies has still proven challenging because of limitations such as the disruption of target antigen expression on CAR-modified T cells, the need to target antigens with limited expression on T cells, and the need for third party donor cells that are either non-alloreactive or have been genome edited at the T cell receptor α constant (TRAC) locus (3). Previous reports suggested CD7-knockout T cells expressing a CD7-specific CAR exhibited antitumor activity in preclinical and clinical trials (3–5). However, the use of anti-CD7 CAR T cells requires knockout CD7 and TRAC expression in cells from third-party donors, and most recipients subsequently relapse. Therefore, we need to find more potential targets for CAR T cell treatment of T cell malignancies.

Adult T-cell leukemia/lymphoma (ATLL) and peripheral T-cell lymphoma (PTCL) are major subtypes of T-cell lymphoma (6). ATLL is a malignancy of mature T lymphocytes that is triggered by human T-cell lymphotropic virus type I (HTLV-1) (7, 8). ATLL was proposed to have four clinical subtypes from ATLL patient databases: acute, lymphoblastic, chronic and smoldering. The 4-year overall survival (OS) rates of ATLL patients are 11%, 16%, 36% and 52% for patients with the acute, lymphoblastic, chronic and smoldering subtypes, respectively (9). Despite the prognosis and recent progress in treatment modalities for patients with acute and lymphoblastic ATLL, their 4-year OS rates are still poor. Moreover, further prolonging the overall survival of ATLL patients with chemotherapy is difficult (10). In addition, ATLL cells express CD3, CD4, and CD25 but lack CD7 (11, 12). Therefore, anti-CD7 CAR T cells are not suitable for ATLL patients.

Expression of the chemokine receptor CCR8, a G protein-coupled receptor (GPCR), is induced by the CC chemokine CCL1/I309 (13, 14). Pervious study suggested that tumor infiltrating Treg expressed high expression of CCR8, whereas NK cells, CD8^+^ T cells, myeloid cells, γδT cells, the bulk of CD4^+^ Tconv cells, and Treg cells found in peripheral blood did not express CCR8 (15). CCR8 controls the immunosuppressive function of tumor infiltrating Treg cells (16), and blocking CCR8 depletes Treg cells and improves antitumor immune responses (17). CCR8 is expressed on ATLL-derived cells and inhibits ATLL cell apoptosis (18). Therefore, we investigated whether targeting CCR8 with CAR T cells significantly suppresses tumor progression in ATLL models. Here, we found that ATLL patients and ATLL cells both expressed CCR8 but not CD7. Moreover, we generated two anti-CCR8 CAR T cell lines and found that anti-CCR8 CAR T cells did not exhibit impaired expansion *in vitro*. In addition, anti-CCR8 CAR T cells exhibited antitumor activity against ATLL cells and CCR8-expressing T-ALL cells, and prolonged the survival of ATLL and Jurkat tumor-bearing mouse models.

## Materials and Methods

### RNA Sequence Analysis

The GEO data set (GEO33615, logFC> 1) used is from GEO database (https://www.ncbi.nlm.nih.gov/geo/), and the download data format is MINIML. Box plots are drawn by boxplot; PCA graphs are drawn by R software package ggord; The box plot is implemented by the R software package ggplot2; the heat map is displayed by the R software package pheatmap (19).

### Chimeric Antigen Receptor Constructs and Lentivirus Production

Third-generation anti-CD19, anti-CCR8(10) (PCT/JP2019/051603) and anti-CCR8 (19) (PCT/JP2019/051603) CAR vectors incorporating CD28, TLR2 (20, 21) and CD3ζ signaling domains were constructed. The sequence of TLR2 domains: CAGGCCAAAAGGAAGCCCAGGAAAGCTCCCAGCAGGAACATCTGCTATGATGCATTTGTTTCTTACAGTGAGCGGGATGCCTACTGGGTGGAGAACCTTATGGTCCAGGAGCTGGAGAACTTCAATCCCCCCTTCAAGTTGTGTCTTCATAAGCGGGACTTCATTCCTGGCAAGTGGATCATTGACAATATCATTGACTCCATTGAAAAGAGCCACAAAACTGTCTTTGTGCTTTCTGAAAACTTTGTGAAGAGTGAGTGGTGCAAGTATGAACTGGACTTCTCCCATTTCCGTCTTTTTGATGAGAACAATGATGCTGCCATTCTCATTCTTCTGGAGCCCATTGAGAAAAAAGCCATTCCCCAGCGCTTCTGCAAGCTGCGGAAGATAATGAACACCAAGACCTACCTGGAGTGGCCCATGGACGAGGCTCAGCGGGAAGGATTTTGGGTAAATCTGAGAGCTGCGATAAAGTCC. Lentiviral particles were produced in HEK-293T cells following polyethyleneimine (Polysciences, Inc., USA)-mediated transfection with the pWPXLd-based transfer plasmid and the packaging and envelope plasmids psPAX2 and pMD2.G. Lentivirus-containing supernatant was harvested at 24, 48, and 72 h post transfection and filtered through a 0.22-µm filter.

### Isolation, Transduction, and Expansion of Primary Human T Lymphocytes

Peripheral blood mononuclear cells (PBMCs) were isolated from healthy adult donors using Lymphoprep (Stem Cell Technologies, Vancouver, Canada). T cells were negatively selected from PBMCs using a MACS Pan T Cell Isolation Kit (Miltenyi Biotec, Bergisch Gladbach, Germany) and activated using microbeads coated with anti-human CD3, anti-human CD2 and anti-human CD28 antibodies (Miltenyi Biotec, Bergisch Gladbach, Germany) at a bead:cell ratio of 1:2 and a density of 2.5×10^6^ cells/ml for two days in RPMI-1640 medium supplemented with 10% heat-inactivated fetal bovine serum (FBS), 100 IU/ml recombinant human IL-2, 10 mM HEPES, 2 mM glutamine and 1% penicillin/streptomycin. On Day 2 post activation, T cells were transduced with CAR vector lentiviral supernatants in the presence of 8 μg/mL polybrene at a multiplicity of infection (MOI) of 2.0 (Sigma–Aldrich, St. Louis, USA). Twelve hours after transduction, T cells were cultured in fresh medium containing IL-2 (300 U/mL); subsequently, fresh medium was added every 3 days to maintain the cell density at approximately 1 × 10^6^ cells/ml. T cells were transduced with the CAR-expressing lentiviral vectors for 24 h. The healthy PBMC donors provided informed consent for the use of their samples for research purposes, and all procedures were approved by the Research Ethics Board of the Guangzhou Institutes of Biomedicine and Health, Chinese Academy of Sciences (GIBH).

### Cells and Culture Conditions

HEK-293T cells were maintained in Dulbecco’s modified Eagle’s medium (DMEM) (Gibco, Grand Island, NY, USA). Cell lines such as Jurkat/Jurkat-GL (T-acute lymphoblastic leukemia), Molt-4 (T-acute lymphoblastic leukemia), MT-4/MT-4-GL (adult T-cell leukemia/lymphoma), and C8166/C8166-GL (adult T-cell leukemia/lymphoma), were maintained in RPMI-1640 medium. The medium was supplemented with 10% heat-inactivated FBS (Gibco, Grand Island, NY, USA), 10 mM HEPES, 2 mM glutamine (Gibco, Grand Island, NY, USA) and 1% penicillin/streptomycin (Gibco, Grand Island, NY, USA). All cells were cultured at 37°C in an atmosphere of 5% carbon dioxide.

### GFP-2A-Luciferase (GL) Generation of Tumor Cells

The GFP-2A-Luciferase (GL) vector contains EGFP, Luciferase and 2A sequence and clones by Sangon Biotech company (Shanghai, China). GL-Lentiviral particles were produced in HEK-293T cells following polyethyleneimine (Polysciences, Inc., USA)-mediated transfection with the pWPXLd-based transfer plasmid and the packaging and envelope plasmids psPAX2 and pMD2.G. Lentivirus-containing supernatant was harvested at 24, 48, and 72 h post transfection and filtered through a 0.22-µm filter. 1 × 10^6^ Tumor cells transduced with 10 ml GL-lentiviral particles for 12 h, and the GFP percentage of GL-tumor cells were detected through flow cytometry for 48 h. GL-tumor cells were sort by FACS Aria. The purity of GL-tumor cells were >95% for killing assay.

### Flow Cytometry

Flow cytometry was performed on a Fortessa cytometer (BD Biosciences, San Jose, CA), and data were analyzed using FlowJo software (FlowJo, LLC, Ashland, OR, USA). The antibodies used, including anti-human CD3-PE (UCHT1), anti-human CD4-APC-Cy7 (OKT4), anti-human CD8-PE_Cy7 (OKT8), anti-human CCR8-APC (SA214G2), and anti-human CD7-FITC (4H9/CD7), were purchased from Biolegend (San Diego, USA). Staining was performed on ice for 30 minutes, and cells were then washed with PBS containing 2% FBS before cytometric analysis. For intracellular staining, cells were fixed and permeabilized with a Foxp3/Transcription factor staining kit (Cat#421403, Biolegend), followed by staining with transcription factor-specific antibodies, such as Tbet, Gata3 and Foxp3. Cells were gently vortexed and were then incubated in the dark for 30 minutes at room temperature. Afterward, the cells were washed once more with cold flow buffer and were then analyzed immediately.

### Cytotoxicity Assays

MT-4-GL, C8166-GL and Jurkat-GL target cells were incubated with 1928z, C1028z or C1928z T cells at the indicated ratio in triplicate wells of U-bottom 96-well plates. Target cell viability was monitored 18 hours later by adding 100 µl/well of the substrate D-luciferin (potassium salt) (Cayman Chemical, Michigan, USA) at 150 µg/mL. Background luminescence was negligible (<1% of the signal from wells containing viable target cells alone). The percentage of viable target cells (%) was calculated as (experimental signal- blank signal)/(targeted signal-blank signal) ×100, and the percentage of cytotoxicity was calculated as 100 – percentage of viable target cells.

### Cytokine Release Assays

T cells were cocultured with target cells at an E:T ratio of 4:1 for 24 hours, and supernatants were analyzed for cytokine release by enzyme-linked immunosorbent assay (ELISA) according to the manufacturers’ protocols. ELISA kits for IFN-γ and Granzyme-B were purchased from Thermo Fisher Scientific Inc., USA.

### Xenograft Models and *In Vivo* Experiment

Animal experiments were performed in the Laboratory Animal Center of GIBH, and all animal procedures were approved by the Animal Welfare Committee of GIBH. All protocols were approved by the relevant Institutional Animal Care and Use Committee (IACUC). NSI mice (22) were maintained in specific pathogen-free (SPF)-grade cages and provided autoclaved food and water. Mice were randomized into experimental groups of ≥ 4. Direct intravenous (tail vein) injection of the indicated leukemia cells in 200 μl of PBS was performed to establish tumors. At the indicated time for each experiment, 2 × 10^6^ transduced human T (GFP^+^ or CAR^+^) cells in 200 μL of PBS were adoptively transferred into tumor-bearing mice systemically by tail vein injection. Peripheral blood was obtained by retro-orbital bleeding. Body weight was measured every 2–3 days as indicated. Tumors were measured every 3 days with a caliper. Tumor volume was calculated using the following formula: (length×width^2^)/2. *In vivo* whole-body imaging of luciferase-labeled cells was performed using a cooled CCD camera system (IVIS 100 Series Imaging System, Xenogen, Alameda, CA, USA). D-luciferin Firefly, potassium salt was injected at 75 mg/kg. Mice were imaged 5 minutes after injection of the substrate. Quantification of the total and average emission was performed using Living Image software.

### Statistical Analysis

Statistical significance was determined using Student’s t test (two groups) or ANOVA with Tukey’s multiple comparison test (three or more groups). All statistical analyses were performed using Prism software, version 7.0 (GraphPad, Inc., San Diego, CA, USA). The gene distribution of GEO database was analyzed using wilcox tests. Kaplan-Meier survival curves of vivo experiments were analyzed using log-rank. P values < 0.05 were considered statistically significant, and the following annotations were used: *, P < 0.05; **, P < 0.01; and ***, P < 0.001. For assessment of differential gene expression, a minimum fold change of 2 was used, and a false discovery rate-corrected P value of < 0.05 (Fisher’s combined p value method) was considered significant (23).

## Results

### CCR8 Was Highly Expressed in ATLL Patients and Cell Lines

**Figure 1:**
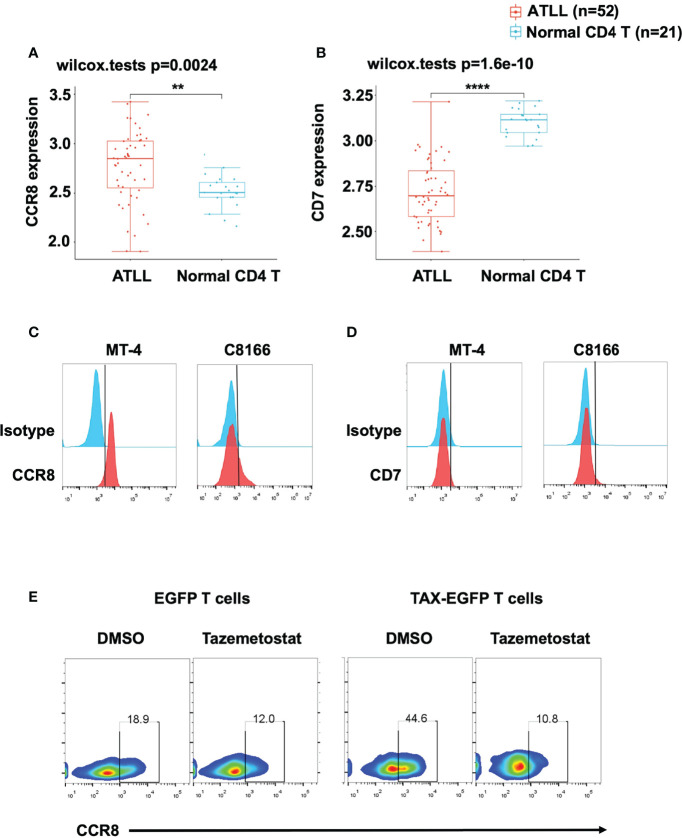
CCR8 was highly expressed in ATLL patients and cell lines. **(A, B)** The expression distribution of CCR8 **(A)** and CD7 **(B)** gene in in ATLL patients (*N* = 52) and normal CD4 T cells (*N* = 21) from healthy donors were analyzed in the publicly datasets (www.aclbi.com) (19) and the data were obtained from GEO database (GSE33615), where the horizontal axis represents different groups of samples, the vertical axis represents the gene expression distribution, where different colors represent different groups, and the upper left corner represents the significance p-value test method (wilcox.tests); **(C, D)** The levels of CCR8 **(C)** and CD7 **(D)**, as detected by flow cytometry, in MT-4 and C8166 cells; **(E)** The level of CCR8 was determined by flow cytometry in TAX-expressing and EGFP T cells (Day 9) upon treatment with DMSO or the EZH2 inhibitor tazemetostat (1 μM, MCE) for 24 h. **P < 0.01, ****P ≤ 0.0001.

To evaluate CCR8 and CD7 expression in primary ATLL patients, we downloaded GSE33615 from GEO database to compare CCR8 and CD7 expression between ATLL patients and normal CD4 T cells. After standardized the GEO data (GSE33615) (**Figure S1A**), We performed principal component analysis (PCA) to confirm the data for subsequent analysis (**Figure S1B**). Interestingly, we found that cells from ATLL patients had higher expression of CCR8 (**Figure 1A**) and lower expression of CD7 (**Figure 1B**) than normal CD4 T cells. In addition, we detected CCR8 expression in two ATLL cell lines, MT-4 and C8166 cells, through flow cytometry. These two ATLL cell lines expressed CCR8 (**Figure 1C**) but did not express CD7 (**Figure 1D**). Given that CCR8 is a new target, we further confirm that CCR8 whether expressed in normal human tissues/cells. Based on publicly available GEPIA database, CCR8 expression levels in human normal tissue are undetectable or very low, compared with the corresponding tumor tissues (**Figures S2A, B**). A previous study suggested that overexpression of TAX, an oncogene in ATLL, promoted primary PBMC expansion *in vitro* (24). Therefore, we further examined the relationship between CCR8 and ATLL. We established a series of TAX-expressing lentiviral vectors and transduced them into primary T cells. We found that compared with EGFP-expressing T cells, TAX-expressing T cells upregulated CCR8 expression (**Figure 1E**). In addition, tazemetostat, an inhibitor of EZH2, suppressed CCR8 expression in TAX-expressing T cells (**Figure 1E**), which was consistent with the finding that EZH2 was associated with ATLL cell development and interacted with TAX (24). Therefore, these results suggest that CCR8 is upregulated in ATLL primary tissues and cell lines and that CCR8 may be a potential therapeutic target for the patients with ATLL.

### Anti-CCR8 CAR T Cells Did Not Impair T Cell Expansion *In Vitro*

**Figure 2:**
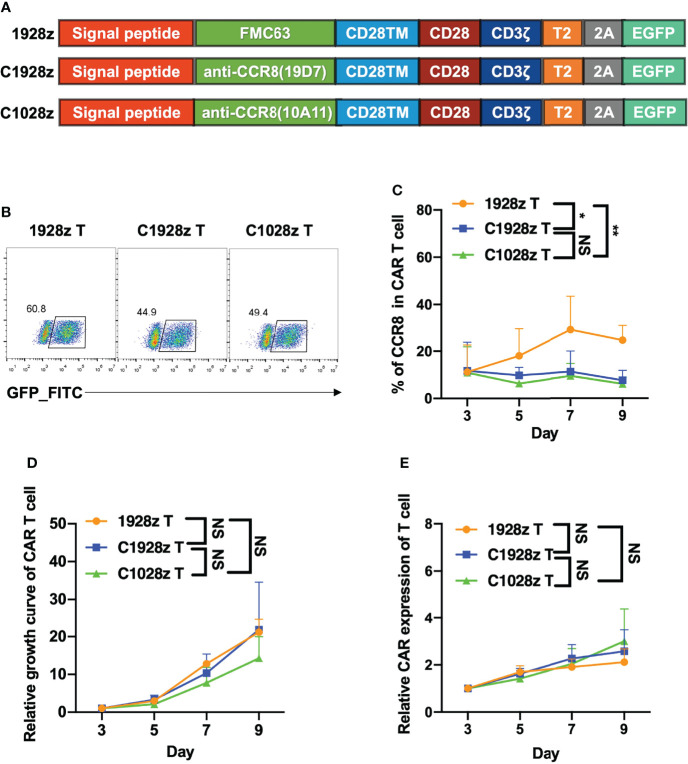
Anti-CCR8 CAR T cells did not impair T cell expansion *in vitro*. **(A)** Anti-CD19 CAR vector based on an anti-CD19 scFv (FMC63, 1928z) and two anti-CCR8 CAR vectors based on an anti-CCR8 scFv (10A11, C1028z) and an anti-CCR8 scFv (19D7, C1928z). All vectors contained expression cassettes encoding a human CD8 leader signal peptide, the CD28, CD3ζ, TLR2 signaling domains, and EGFP fused to the sequences described in **Figure 2A**; **(B)** CAR expression on CAR T cells was detected by flow cytometry; **(C)** CCR8 expression on CAR T cells was detected by flow cytometry at the indicated time for three different donors; **(D)** The relative growth of CAR T cells was analyzed by flow cytometry at the indicated time for three different donors; **(E)** The CAR level of CAR T cells were detected by flow cytometry at the indicated time for three different donors. **(C–E)** Data are shown as the mean ± SEM values; two-way ANOVA with Tukey’s multiple comparisons test; *P < 0.05, **P ≤ 0.01. n.s >0.05.

To investigated whether anti-CCR8 CAR T cells suppress T cell function *in vitro*, we constructed two third-generation CAR vectors, namely, anti-CCR8 (10A11) C1028z and anti-CCR8 (19D7) C1928z, and anti-CD19 CAR (1928z) served as negative control, which contain the scFv, the human CD28 transmembrane domain (CD28TM) and endodomain, the human CD3ζ T cell activating domain, a human TLR2 domain (T2) and EGFP; these vectors were introduced into human T cells through lentiviral transduction (**Figures 2A, B**). We found that compared with that in the 1928z group, the CCR8 levels in the C1028z and C1928z groups were decreased (**Figure 2C**). Furthermore, we found that the T cell expansion and relative CAR expression in the C1028z and C1928z groups were similar to those in the 1928z group (**Figures 2D, E**), which was consistent with pervious study that T cells expressing CD5 CAR undergo only limited fratricide and can be expanded long-term *in vitro (* 25). In addition, we found the CD4/CD8 ratio and TH cell classification in the C1928z and C1028z groups were similar to those in the 1928z group (**Figures S3A–C**). These results suggest that anti-CCR8 CAR T cells do not impair T cell expansion *in vitro*.

### Anti-CCR8 CAR T Cells Exhibited Antitumor Efficacy Against ATLL Cells *In Vitro*

**Figure 3:**
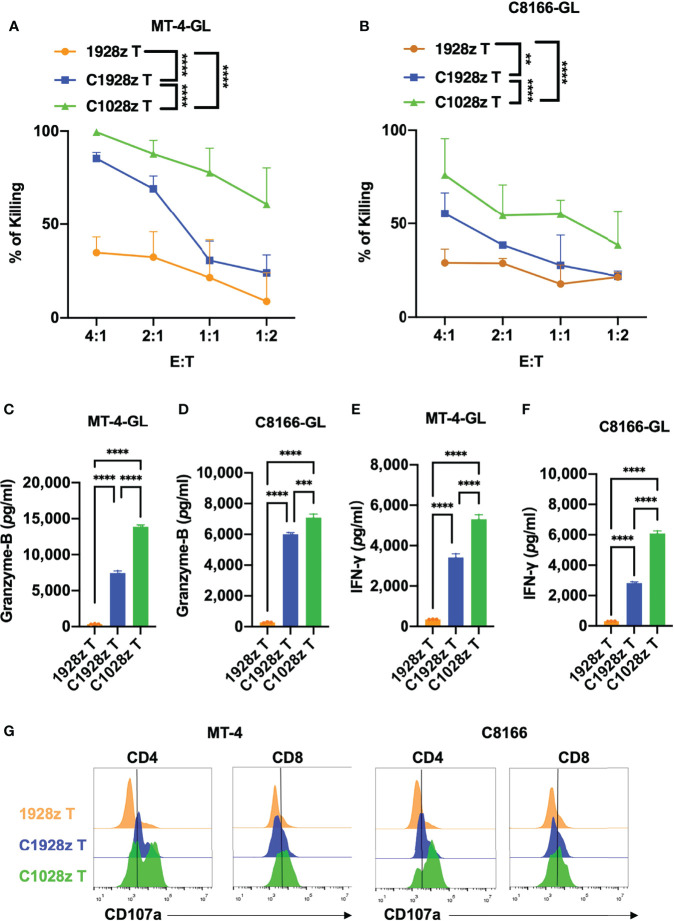
Anti-CCR8 CAR T cells exhibited antitumor efficacy against ATLL cells *in vitro*. **(A, B)** The percentages of MT-4 **(A)** and C8166 cells **(B)** whose lysis was induced by 1928z, C1928z and C1028z T cells; **(B–F)** 1928z, C1928z and C1028z T cells were incubated with MT-4 or C8166 cells at a 4:1 effector **(E)** target (T) ratio in 96-well round-bottom plates for 24 hours at 37°C. Supernatants were then harvested and analyzed with a multiplex immunoassay to determine the concentrations of the indicated cytokines. The concentrations of Granzyme-B **(C, D)** and IFN-γ **(E, F)** were measured by ELISA; **(G)** The level of CD107a, as detected by flow cytometry, in 1928z, C1928z and C1028z T cells after coculture with MT-4 or C8166 cells for 24 h; **(A, B)** Data are shown as the mean ± SEM values; two-way ANOVA with Tukey’s multiple comparisons test; **P ≤ 0.01, ****P ≤ 0.0001; **(C–F)** Data are shown as the mean ± SEM values; one-way ANOVA with Tukey’s multiple comparisons test; **P ≤ 0.01, ***P ≤ 0.001.

To further investigate whether anti-CCR8 CAR T cells suppress ATLL cell growth *in vitro*, we performed a cytotoxicity assay with C1028z and C1928z and 1928z T cells in two ATLL cell lines that did not express CD19 (**Figure S4A**), and showed that C1028z and C1928z T cells exhibited higher cytotoxicity than 1928z T cells after coculture with MT-4-GFP-2A-Luciferase (GL) and C8166-GL cells at the indicated effector:target (E:T) ratio *in vitro* (**Figures 3A, B**). Moreover, the C1028z and C1928z groups had higher expression of Granzyme-B (**Figures 3C, D**) and IFN-γ (**Figures 3E, F**) than the 1928z group. In addition, we found that the expression of CD107a (26), a sensitive marker for cytotoxic activity determination, was increased in both the C1028z and C1928z groups compared with the 1928z group (**Figure 3G**). Notably, C1028z T cells exhibited better antitumor efficacy than C1928z T cells against the two ATLL cell lines (**Figures 3A–G**). These collective results suggest that C1028z and C1928z T cells exhibit a considerable antitumor effect on CCR8^+^ ATLL cells *in vitro*.

### Anti-CCR8 CAR T Cells Exhibited Antitumor Efficacy Against ATLL Cells *In Vivo*

**Figure 4:**
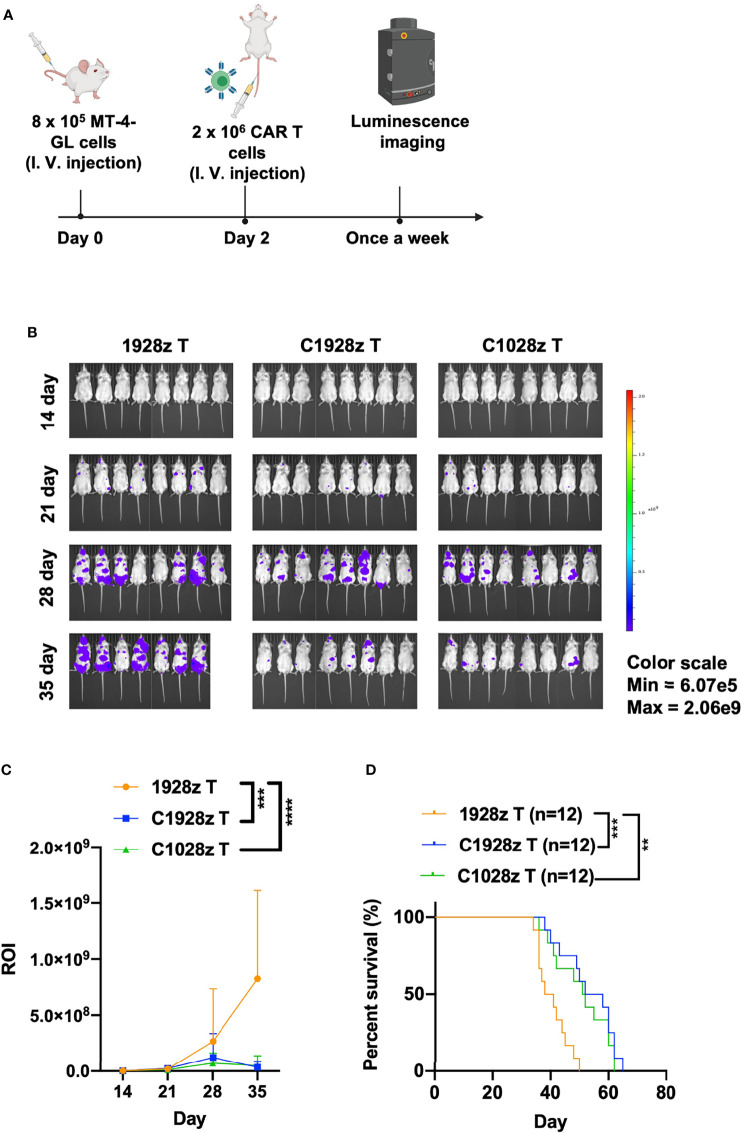
Anti-CCR8 CAR T cells exhibited antitumor efficacy against ATLL cells *in vivo*. **(A)** Schematic representation of the experiments; **(B)** BLI of MT-4-GL mice intravenously injected with MT-4-GL cells and then treated with 1928z, C1928z or C1028z T cells. Briefly, NSI mice received an i.v. injection of 1 × 10^6^ MT-4-GL cells. After 2 days, 2 × 10^6^ 1928z, C1928z or C1028z T cells were intravenously injected into the MT-4-GL NSI model mice (*N* = 8 mice/group), and BLI was conducted on Days 14, 21, 28 and 35. Representative results of one from two repeated experiment are shown (total mice/group = 12); **(C)** Statistical analysis of the ROI of BLI at each time point with two repeated experiments (total mice/group = 12); **(D)** Kaplan-Meier survival analysis of treatment with1928z, C1928z or C1028z T cells were shown with two repeated experiments (total mice/group = 12); **(C)** Data are shown as the mean ± SEM values; two-way ANOVA with Tukey’s multiple comparisons test; ***P ≤ 0.001; ****P ≤ 0.0001; **(D)** Statistical analysis for survival curves represents log-rank test, **P < 0.01, ***P ≤ 0.001.

We next evaluated the antitumor effects of anti-CCR8 CAR T cells *in vivo*. We infused T cells expressing C1028z, C1928z and 1928z into immunodeficient NSI mice (22) that had been inoculated with MT-4-GL cells (**Figure 4A**). C1928z and C1028z T cells induced significant regression of tumors formed from MT-4-GL cells, while the tumors in the 1928z group continued to progress, as detected by bioluminescence imaging (BLI) (**Figures 4B, C**, **S5A**). In addition, C1928z and C1028z T cells prolonged the survival of MT-4 tumor-bearing mice, compared with 1928z T cells (**Figure 4D**). Interestingly, C1028z T cells had higher cytotoxic activity than C1928z T cells against MT-4 cells *in vitro* (**Figure 3**), but the anticancer response of C1028z T cells was similar to that of C1928z T cells *in vivo* (**Figures 4B–D**). Therefore, these results demonstrate that C1028z and C1928z T cells exhibit antitumor effects on CCR8^+^ ATLL cells and prolonged the survival of MT-4-GL tumor-bearing mice models *in vivo*.

### Anti-CCR8 CAR T Cells Exhibited Antitumor Efficacy Against CCR8-Expressing T-ALL Cells

**Figure 5:**
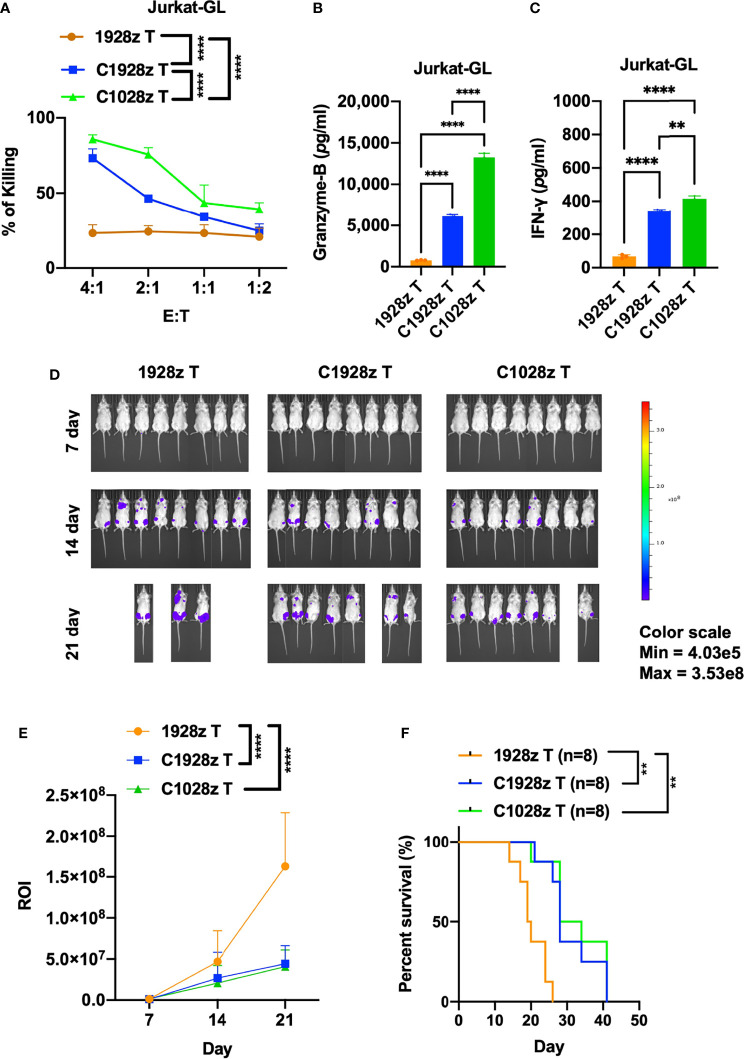
Anti-CCR8 CAR T cells exhibited antitumor efficacy against CCR8-expressing T-ALL cells. **(A)** The percentages of Jurkat cells whose lysis was induced by 1928z, C1928z and C1028z T cells; **(B, C)** 1928z, C1928z and C1028z T cells were incubated with Jurkat cells at a 4:1 effector **(E)**: target (T) ratio in 96-well round-bottom plates for 24 hours at 37°C. Supernatants were then harvested and analyzed with a multiplex immunoassay to determine the concentrations of the indicated cytokines. The concentrations of Granzyme-B **(B)** and IFN-γ **(C)** were measured by ELISA; **(D, E)** BLI of mice intravenously injected with Jurkat-GL cells and then treated with 1928z, C1928z or C1028z T cells. Briefly, NSI mice received an i.v. injection of 1 × 10^6^ Jurkat-GL cells (*N* = 8 mice/group). Representative results of one from two repeated experiment are shown (total mice/group = 12). After 2 days, 2 × 10^6^ 1928z, C1928z or C1028z T cells were injected through the tail vein, and BLI was conducted on Days 7, 14 and 21. **(E)** Statistical analysis of the ROI of BLI at each time point (*N* = 8 mice/group); **(F)** Kaplan-Meier survival analysis of treatment with1928z, C1928z or C1028z T cells were shown (*N* = 8 mice/group). **(A, E)** Data are shown as the mean ± SEM values; two-way ANOVA with Tukey’s multiple comparisons test; **P ≤ 0.01, ****P ≤ 0.0001; **(B, C)** Data are shown as the mean ± SEM values; one-way ANOVA with Tukey’s multiple comparisons test; **P ≤ 0.01, ****P ≤ 0.0001; **(F)** Statistical analysis for survival curves represents log-rank test, **P ≤ 0.01.

Although anti-CD7 CAR T cells were found to exhibit a good safety profile and achieve a high complete remission rate, the presence of CD7-negative tumor cells may lead to relapse. We examined whether anti-CCR8 CAR T cells inhibit the growth of T-ALL cells in addition to ATLL cells. First, we detected CCR8 and CD19 expression on T-ALL cells, including Jurkat and Molt-4 cells. We found that CCR8 was expressed on Jurkat cells but not Molt-4 cells (**Figure S6A**), and CD19 did not expressed on Jurkat and Molt-4 cells (**Figure S6B**). Moreover, we found that C1028z and C1928z T cells exhibited higher cytotoxicity than 1928z T cells after coculture with Jurkat cells at the indicated effector to target (E:T) ratio *in vitro* (**Figure 5A**), but C1028z and C1928z T cells did not suppress the growth of Molt-4 *in vitro* (**Figure S6C**). In addition, we found that T cells expressing C1028z and C1928z produced higher levels of cytotoxic cytokines, such as Granzyme-B (**Figure 5B**) and IFN-γ (**Figure 5C**), than T cells expressing 1928z. We further evaluated the antitumor effects of anti-CCR8 CAR T cells in the Jurkat NSI mouse model. We infused T cells expressing C1028z, C1928z and 1928z into Jurkat NSI model mice. We found that compared with 1928z T cells, C1928z and C1028z T cells significantly suppressed the growth of Jurkat tumors *in vivo* and improved the overall-survival of Jurkat tumor-bearing mice (**Figures 5D–F**, **S7A–C**). Therefore, these collective results suggest that C1928z T cells and C1028z T cells can suppress the growth of CCR8-expressing T-ALL tumors and improve the overall survival of tumor-bearing mice.

## Discussion

Adult T-cell leukemia/lymphoma (ATLL) patients have an extremely poor prognosis that cannot be prolonged through chemotherapy alone. Here, we show that activated human T cells that express the CCR8 CAR can specifically recognize and kill ATLL cells *in vitro* (**Figures 3A, B**). Moreover, anti-CCR8 CAR T cells produced higher levels of IFN-γ, Granzyme-B and CD107α than 1928z T cells after coculture with ATL cells (**Figures 3C–F**). In addition, anti-CCR8 CAR T cells significantly suppressed MT-4 tumor progression *in vivo* and prolonged the survival of MT-4 tumor-bearing mice (**Figure 4**). Similar to T cells transduced with a CD5 CAR, anti-CCR8 CAR T cells did not significantly suppress T cell expansion *in vitro*. In addition, CCR8 was largely expressed on tumor infiltrating Treg cells, indicating that anti-CCR8 CAR T cells did not impair their anticancer immune response. Therefore, these results suggest that anti-CCR8 CAR T cells exhibit stronger antitumor immune responses in CCR8^+^ ATLL or T-ALL.

Although the success of CD19 CAR T cell therapy in B-ALL has revolutionized anticancer therapy, the high rate of complete response is sometimes limited by the emergence of CD19-negative leukemia cells (27). Here, we found that CCR8 is also expressed on T-ALL cells such as Jurkat cells (**Figure S6A**). Furthermore, we showed that anti-CCR8 CAR T cells inhibited the growth of Jurkat cells *in vitro* (**Figure 5A**). In addition, the production of IFN-γ and Granzyme-B was improved in the C1928z and C1028z groups compared with the 1928z group after coculture with Jurkat cells (**Figures 5B, C**). Notably, anti-CCR8 CAR T cells reduced Jurkat tumorigenesis and prolonged survival of Jurkat tumor-bearing mice (**Figures 5D–F**). Therefore, anti-CD7 and anti-CCR8 dual CAR T cells may be a good choice to prevent antigen escape and further improve the antitumor effect of CCR8^+^ T-ALL treatment. In addition, CCR8 is a driver of Treg cells that secrete immunosuppressive cytokines, such as TGFβ1, to inhibit MSLN CAR T cell function in solid tumors (28). Therefore, anti-MSLN and anti-CCR8 dual CAR T cells could impair Treg cell differentiation and increase the MSLN CAR T cell population in solid tumors.

In conclusion, we demonstrated that anti-CCR8 CAR T cells exhibit a stronger anticancer response than other CAR T cells against CCR8^+^ ATLL cells and T-ALL cells and provided a novel treatment for patients with CCR8^+^ T cell malignances.

## Data Availability Statement

The raw data supporting the conclusions of this article will be made available by the authors, without undue reservation.

## Ethics Statement

The animal study was reviewed and approved by the Animal Welfare Committee of Guangzhou Institutes of Biomedicine and Health, Chinese Academy of Sciences.

## Author Contributions

PTL, DZ, and XW conceived and designed the research. DZ, XW, LC, LQ, ZJ, RZ, YL, JS, QW, YGL, and SW performed *in vitro* assays and animal experiments. PL, DZ, and XW contributed to the writing and final approval of the manuscript and provided financial support. All authors contributed to the article and approved the submitted version.

## Funding

This study was supported by the National Key Research and Development Plan (No. 2021YFE0202800 to PTL), the National Natural Science Foundation of China (Nos. 81961128003 to PTL, 81972672 to PTL, 81773301 to ZJ, 81870121 to PTL, 81873847 to JY, and 32170946 to ZJ), The Youth Innovation Promotion Association of the Chinese Academy of Sciences (No. 2020351 to ZJ), the Guangdong Provincial Significant New Drugs Development (Nos. 2019B020202003 to PTL, 2019A1515010062 to YY, and 2020A1515011516 to XW), the Guangzhou Science and Technology Plan Project (No. 201907010042 to PTL), 2020B1212060052, the Frontier Research Program of the Guangzhou Regenerative Medicine and Health Guangdong Laboratory (No. 2018GZR110105003 to PTL), the Science and Technology Planning Project of Guangdong Province, China (2020B1212060052), the Science and Technology Program of Guangzhou, China (No. 202002020083 to XL), the Open Project of the State Key Laboratory of Respiratory Disease (No. SKLRD-OP-202002 to ZZ), the University Grants Committee/Research Grants Council of Hong Kong (Project No. AoE/M-401/20), and the Innovation and Technology Fund (ITF) from the Hong Kong SAR government, the Youth Talent Promotion project of Guangzhou Association for Science and Technology(No. X20210201015 to LY).

## Conflict of Interest

PTL is a scientific founder of GZI and GZCB and has equity in GZI and GZCB. Author ZT was employed by Guangdong Zhaotai *In vivo* Biomedicine Ltd. and Guangdong Zhaotai Cell Biology Technology Ltd.

The remaining authors declare that the research was conducted in the absence of any commercial or financial relationships that could be construed as a potential conflict of interest.

## Publisher’s Note

All claims expressed in this article are solely those of the authors and do not necessarily represent those of their affiliated organizations, or those of the publisher, the editors and the reviewers. Any product that may be evaluated in this article, or claim that may be made by its manufacturer, is not guaranteed or endorsed by the publisher.
