# Supplementary figures and images for: The Chemokine Receptor CCR8 Is a Target of Chimeric Antigen T Cells for Treating T Cell Malignancies
Source: Front Immunol. 2022 May 26;13:808347. doi: 10.3389/fimmu.2022.808347 (PMC9182403; doi:10.3389/fimmu.2022.808347)

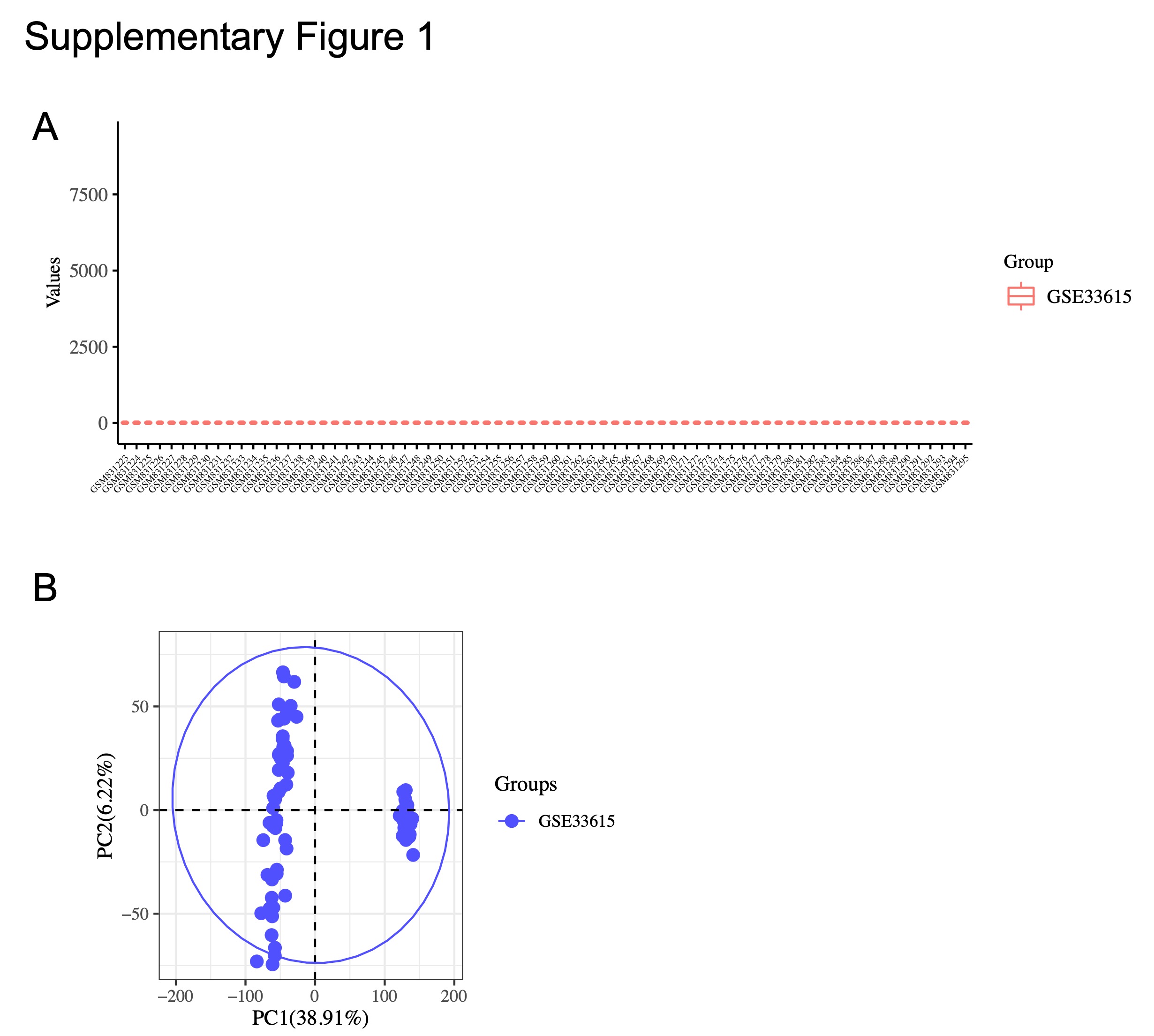

Supplement: Supplementary file 1 [file Image_1.jpeg]

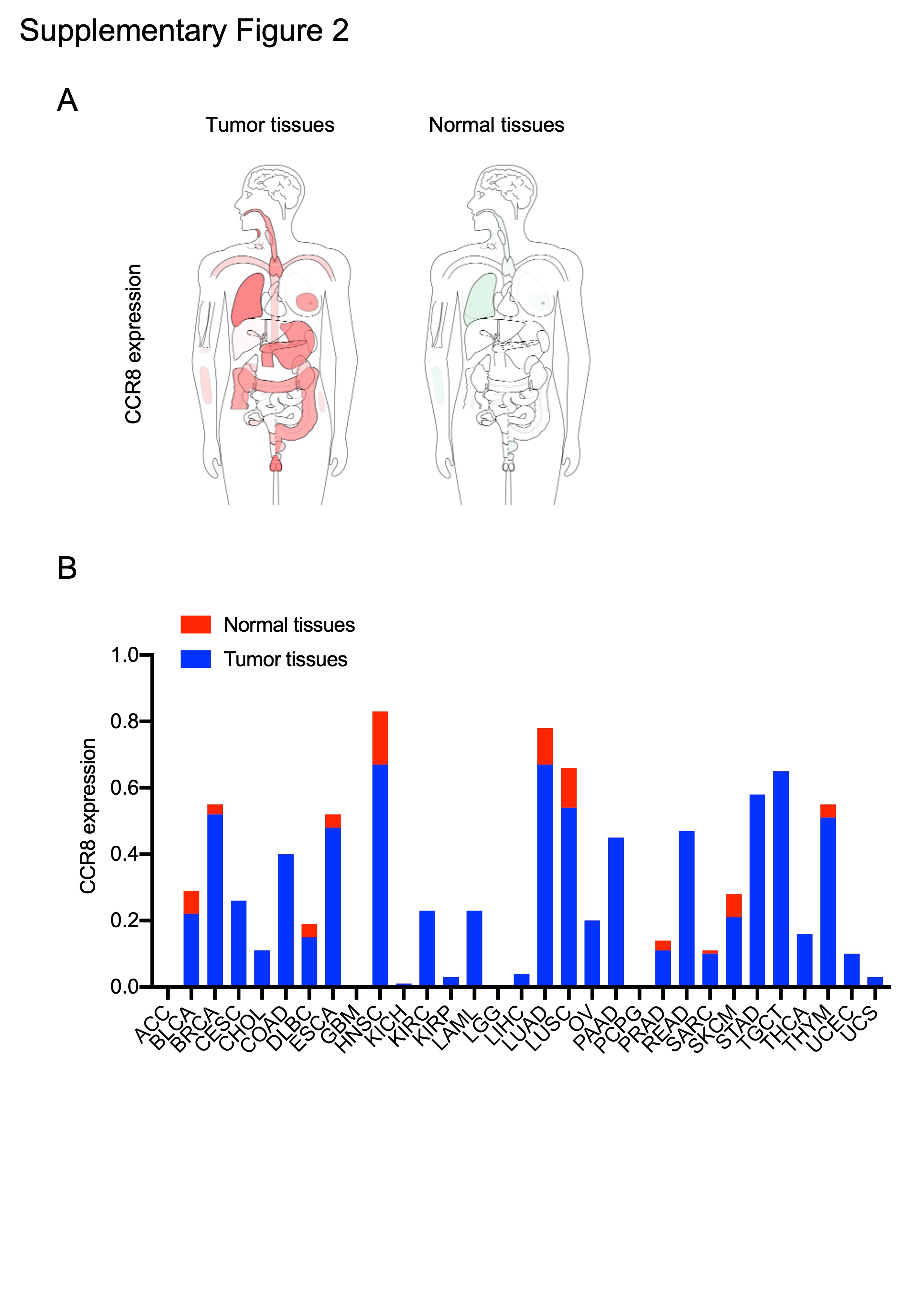

Supplement: Supplementary file 2 [file Image_2.jpeg]

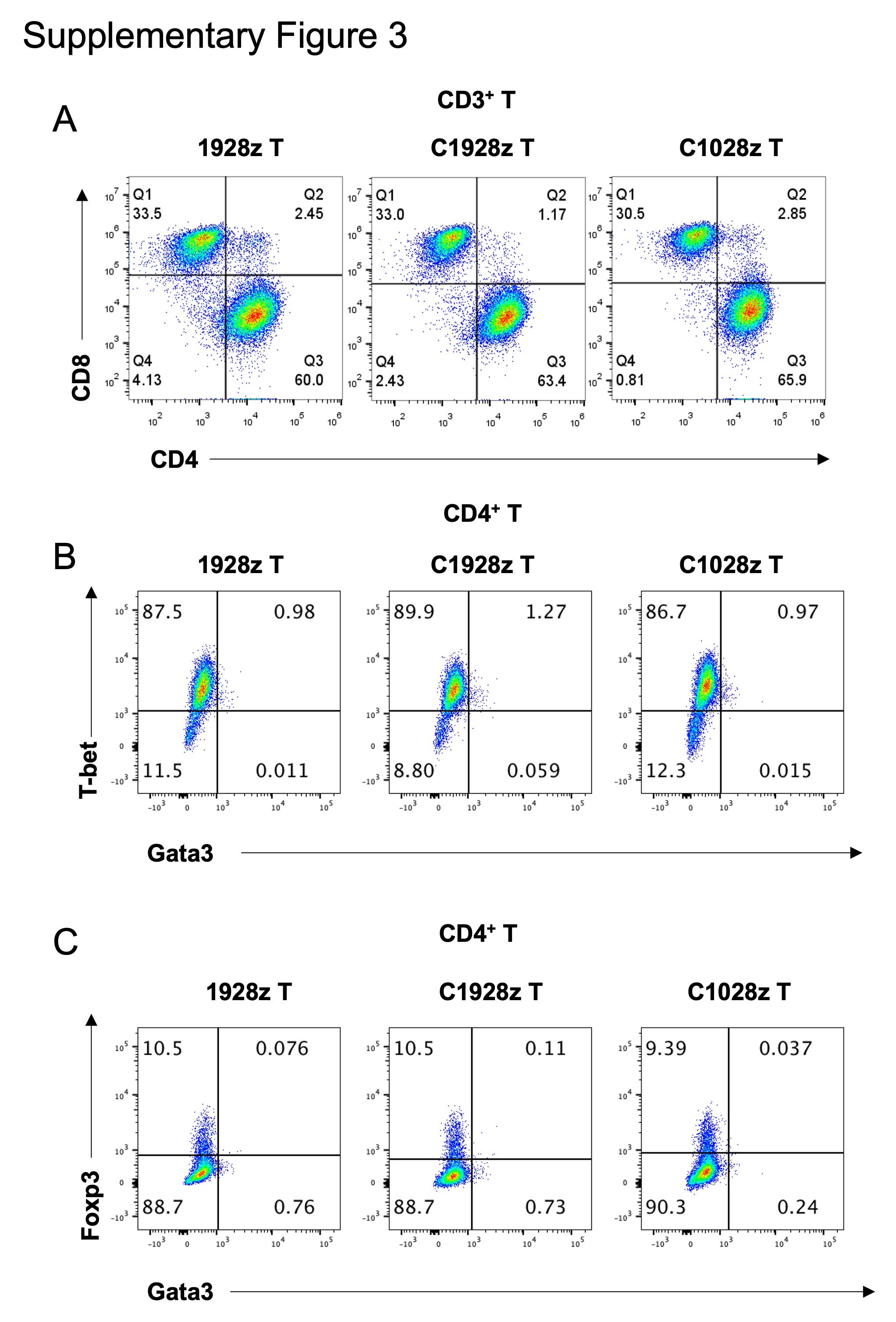

Supplement: Supplementary file 3 [file Image_3.jpeg]

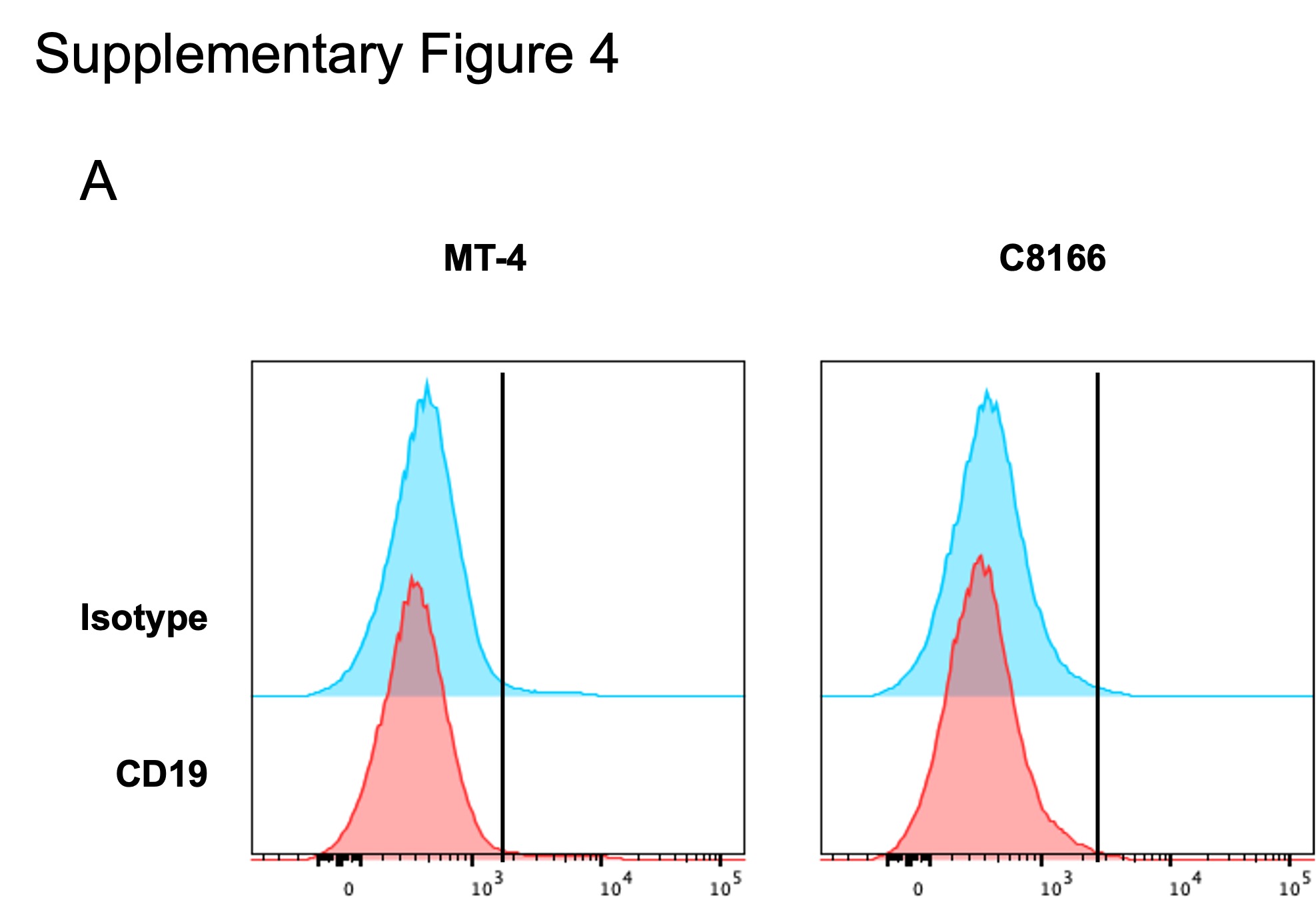

Supplement: Supplementary file 4 [file Image_4.jpeg]

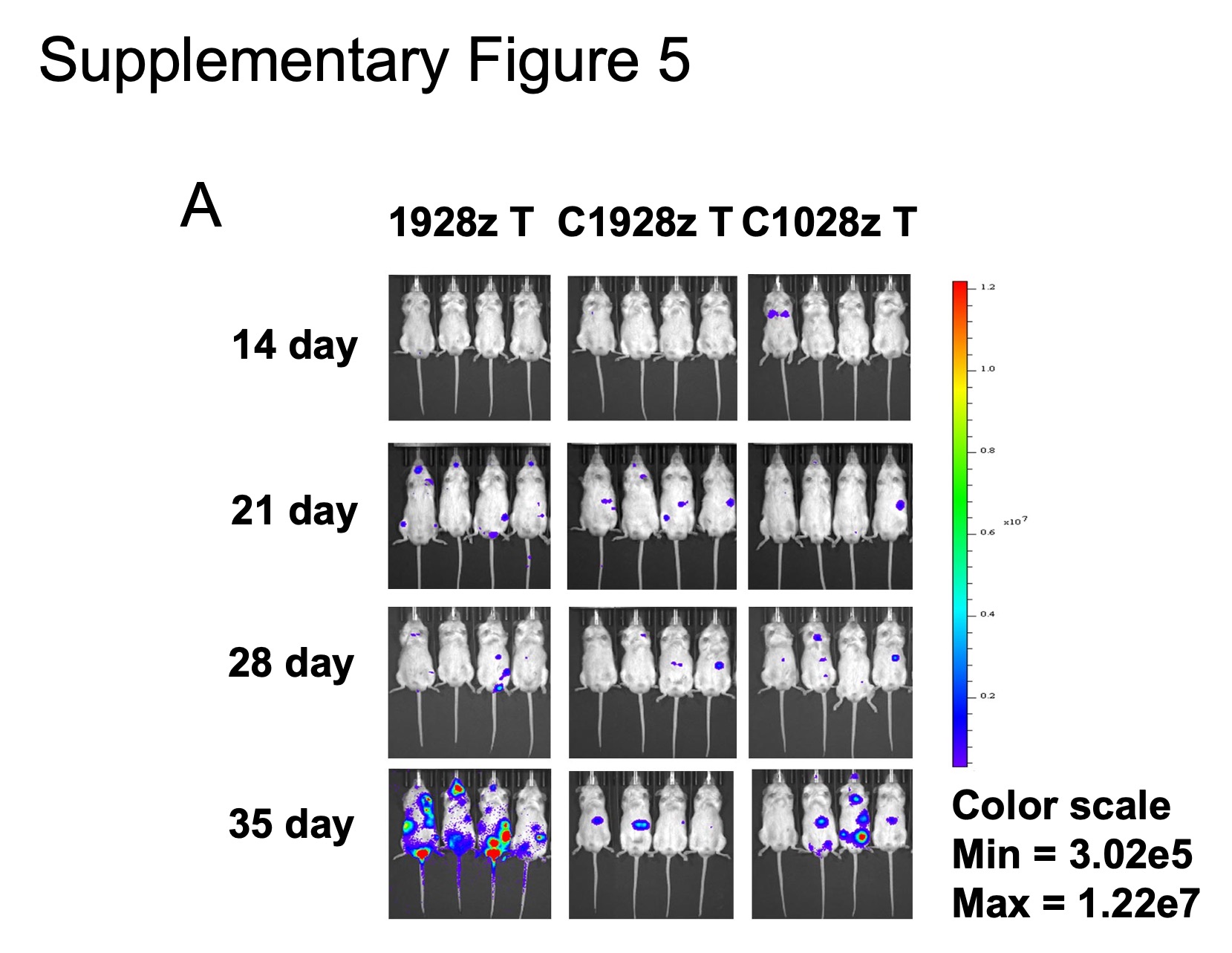

Supplement: Supplementary file 5 [file Image_5.jpeg]

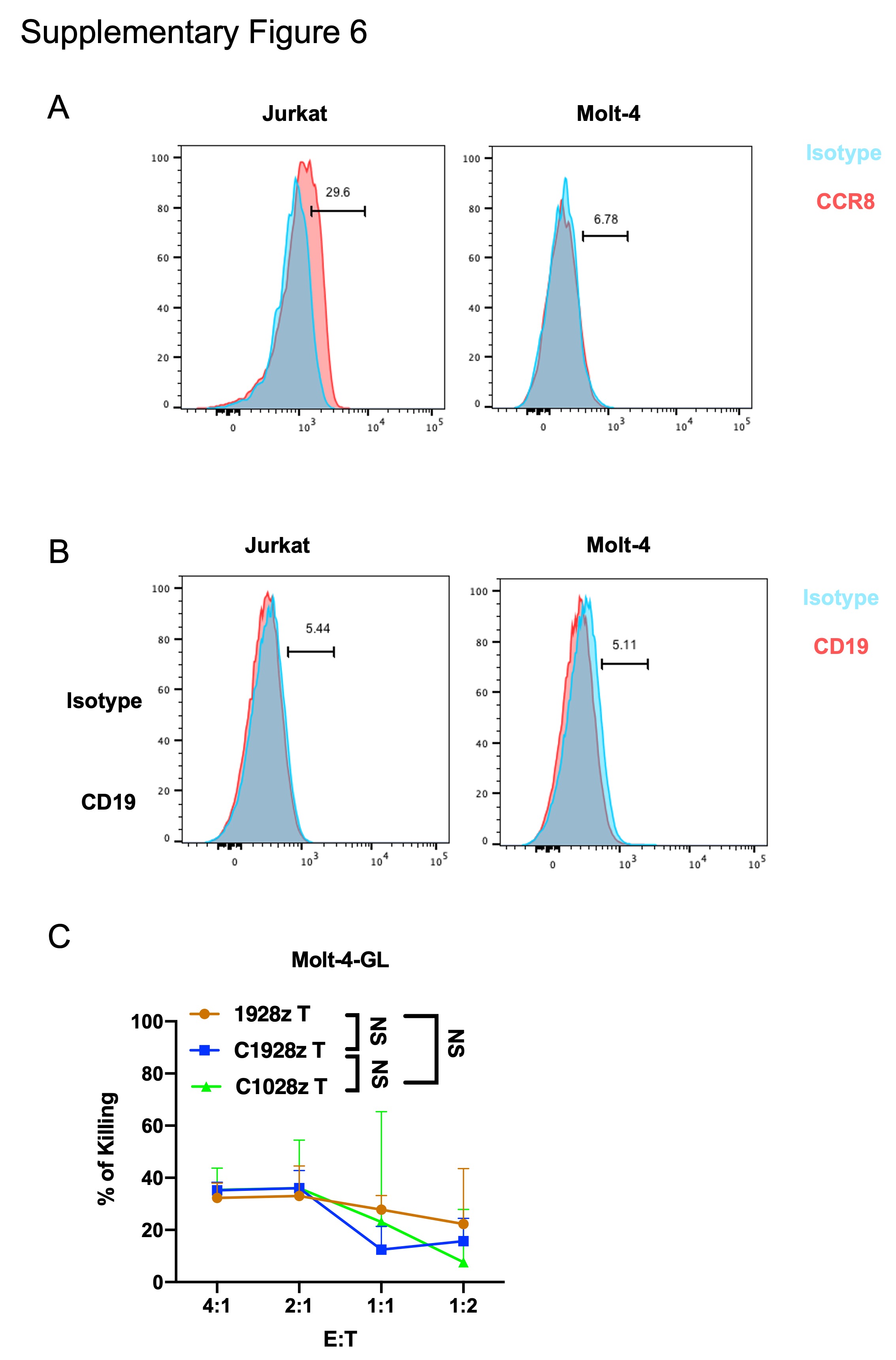

Supplement: Supplementary file 6 [file Image_6.jpeg]

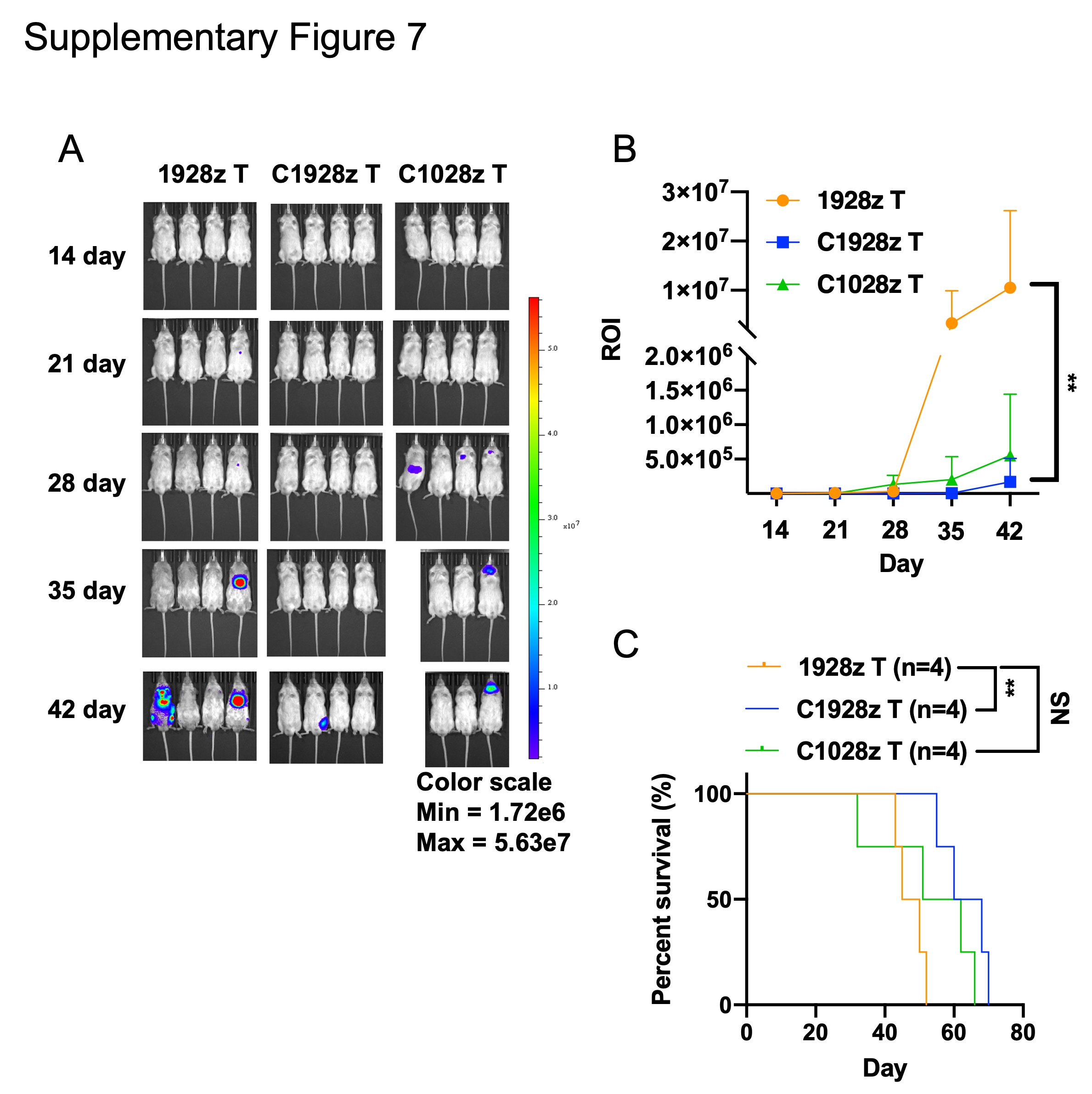

Supplement: Supplementary file 7 [file Image_7.jpeg]
